# Supplementary material for: Pervasive glacier retreats across Svalbard from 1985 to 2023
Source: Nat Commun. 2025 Jan 15;16:705. doi: 10.1038/s41467-025-55948-1 (PMC11735618; doi:10.1038/s41467-025-55948-1)
Supplement: Supplementary file 1 — Supplementary Information [file 41467_2025_55948_MOESM1_ESM.pdf]

Supplementary Material for **Pervasive glacier retreats across Svalbard from 1985 to 2023**

Tian Li[1,2*], Stefan Hofer[1], Geir Moholdt[3], Adam Igneczi[1], Konrad Heidler[2], Xiao Xiang Zhu[2,4], and Jonathan Bamber[1,2]

[1]Bristol Glaciology Centre, School of Geographical Sciences, University of Bristol, Bristol, BS8 1SS, UK
[2]Chair of Data Science in Earth Observation, Department of Aerospace and Geodesy, Technical University of Munich, Munich 80333, Germany
[3]Norwegian Polar Institute, Tromsø, Norway
[4]Munich Center for Machine Learning, Technical University of Munich, Munich 80333, Germany

Corresponding author: tian.li@bristol.ac.uk

Table S1: Linear correlation coefficients between seasonal calving front retreat rates and different time-evolving environmental variables in different sectors for non-surging glaciers with seasonal cycles over the period of 2014-2023. Environmental variables include sea ice concentration (SIC), surface runoff (RU), 3 m air temperature (AT), 20-100 m depth-averaged subsurface ocean temperature (PT). Sectors include including Northeast Spitsbergen (NE), Northwest Spitsbergen (NW), South Spitsbergen (SS), Vestfonna (VF), Austfonna (AF), and Barentsøya and Edgeøya (BE). 'All' means the entire Svalbard.

Sector	SIC R^2	SIC P-value	RU R^2	RU P-value	AT R^2	AT P-value	PT R^2	PT P-value
NE	0.48	0.012	0	0.96	0.35	0.044	0.47	0.014
AF	0.76	0	0.01	0.73	0.34	0.045	0.75	0
BE	0.81	0	0.09	0.35	0.32	0.057	0.79	0
NW	0.17	0.182	0.58	0.004	0.83	0	0.86	0
SS	0.55	0.006	0.22	0.12	0.37	0.036	0.85	0
VF	0.46	0.015	0.18	0.17	0.55	0.006	0.7	0.001
All	0.83	0	0.3	0.07	0.63	0.002	0.97	0

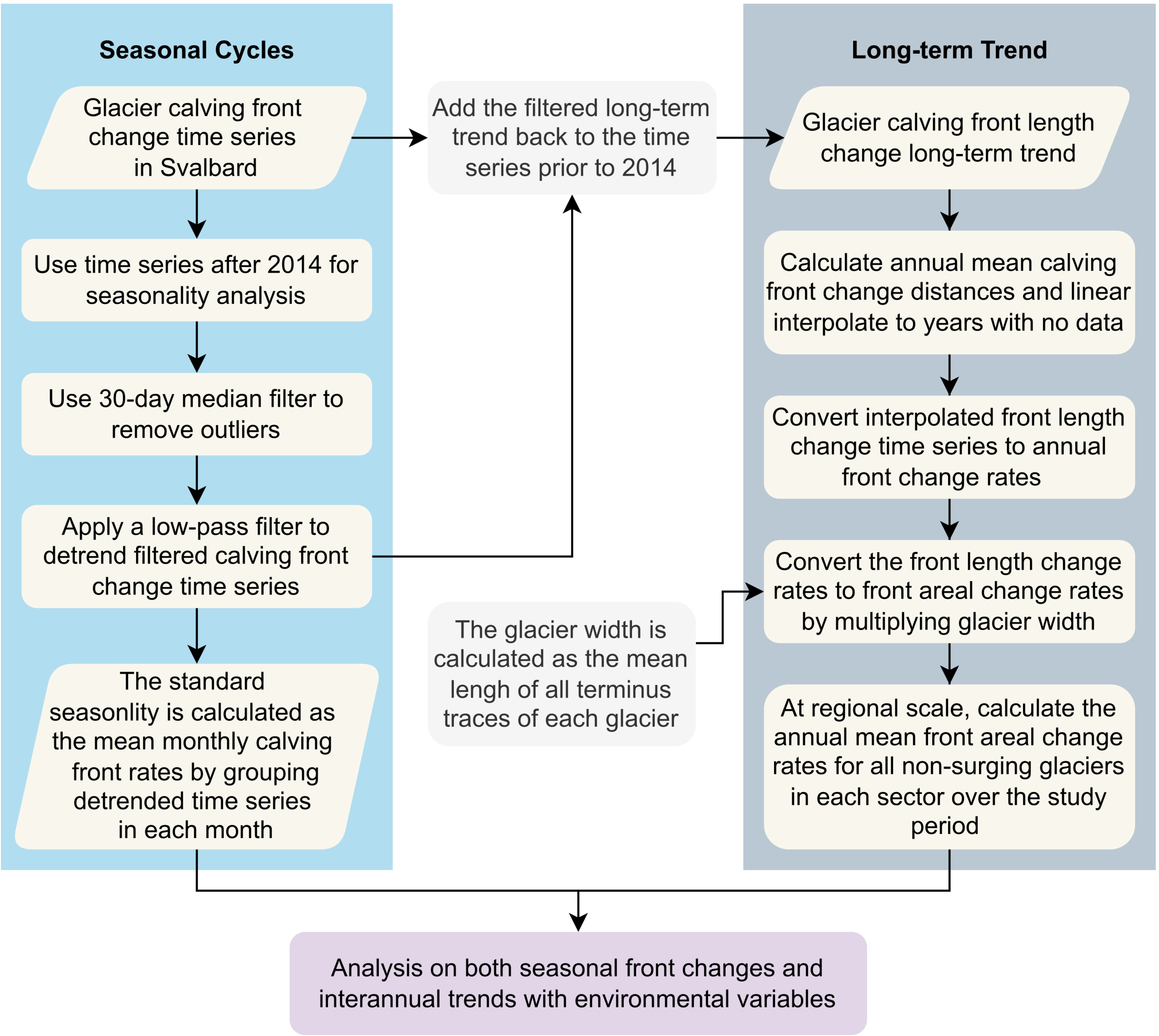

Figure S1: The glacier calving front change time series decomposition workflow for analyzing the seasonal front cycles in 2014-2023 (blue panel) and long-term calving front changes in 1985-2023 (grey panel). The seasonal calving front changes and long-term rates are measured along the glacier centerlines defined in [1]. Note here that only non-surging glaciers are considered when calculating the regional mean values.

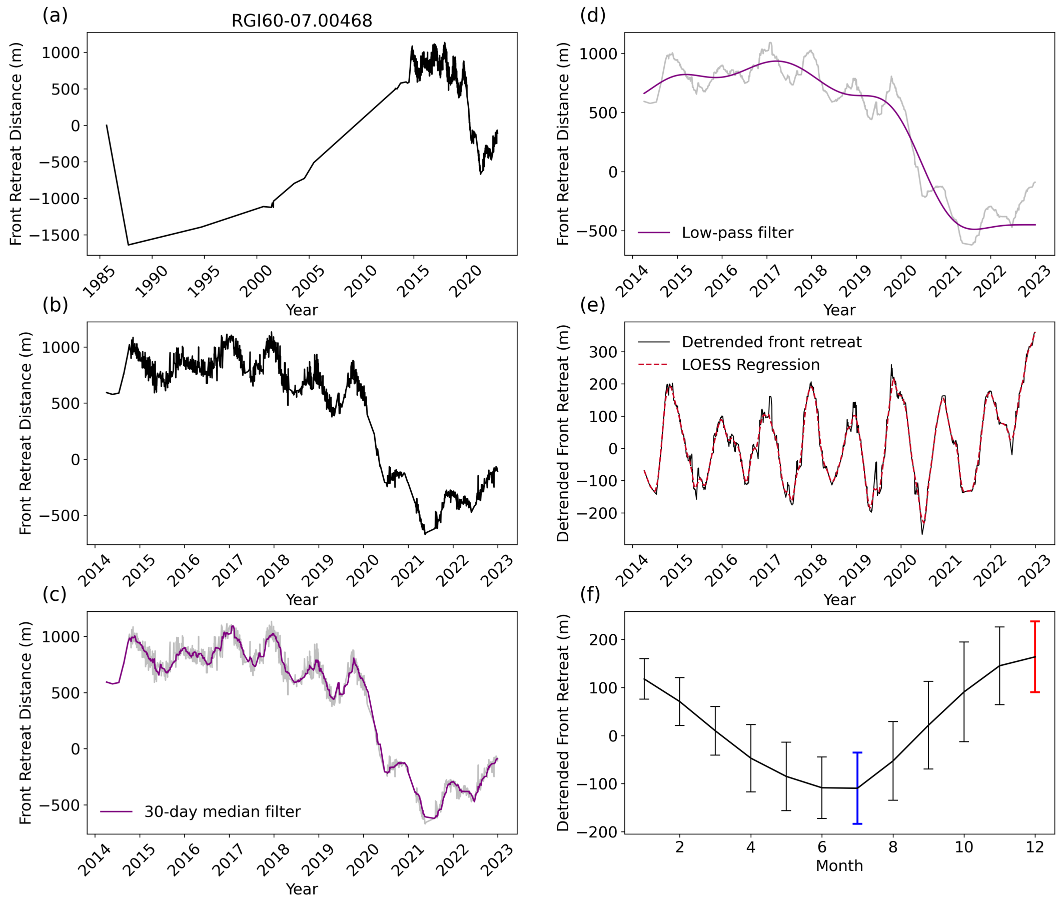

Figure S2: Example of calving front change time series decomposition anlaysis for a surging glacier Vestre Osbornebreen (RGI60-07.00468) following the steps in flowchart (Figure S1). (a) the calving front change time series in 1985-2023. (b) zoomed-in time series in 2014-2023 that is used in calculating the seasonal calving front change rates. (c) Use a 30-day mdeidan filter to remove outliers from the calving front change time series (blue line). (d) apply a low-pass filter (purple line) to detrend the filtered time series (grey line) in (c). The trend will be used in long-term retreat analysis (gery panel in Figure S1). (e) the detrended calving front change results (black line) from (d), here we apply the LOESS regression to allow better visualization of the seasonal cycles (red line). (f) The seasonal retreat rates for each glacier are calculated as the mean detrended monthly calving front rates in each month over the period of 2014-2023. The blue error bar denotes the most advanced month and the red error bar denotes the most retreated month.

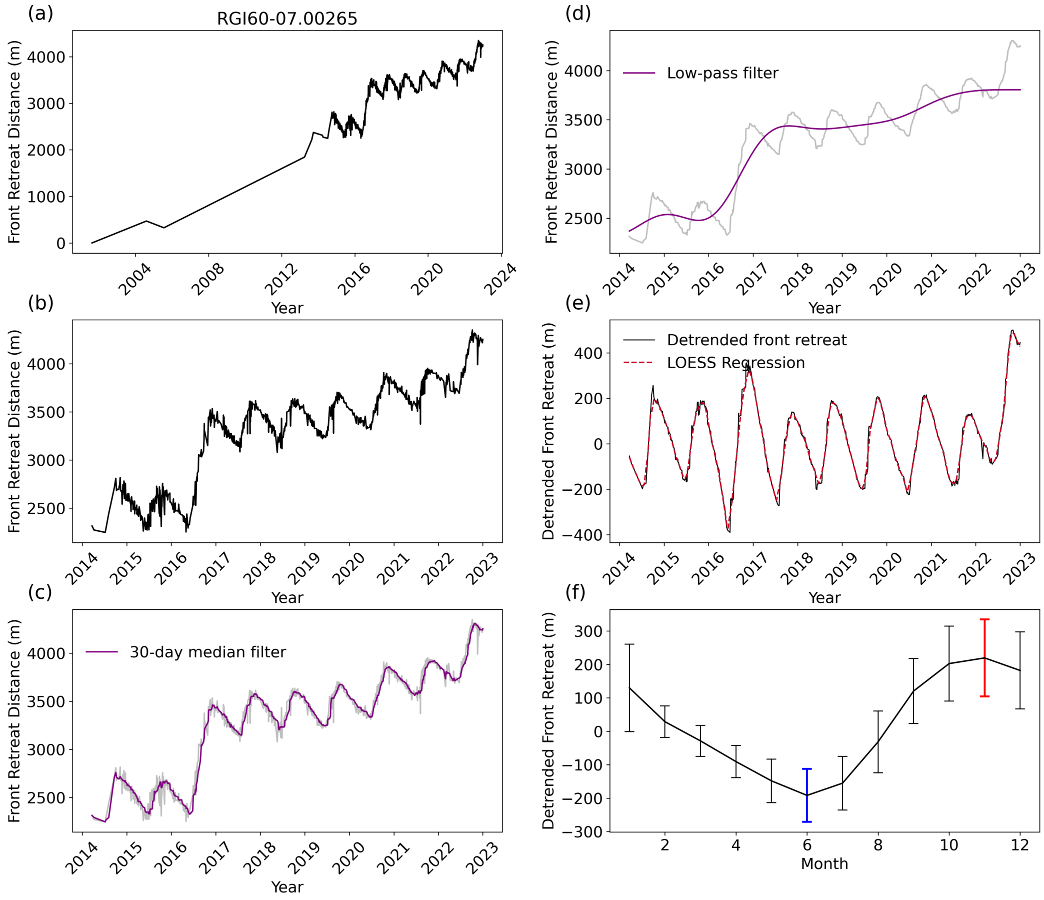

Figure S3: Example of calving front change time series decomposition anlaysis for a retreating glacier Olsokbreen (RGI60-07.00265) following the steps in flowchart (Figure S1). (a) the calving front change time series in 1985-2023. (b) zoomed-in time series in 2014-2023 that is used in calculating the seasonal calving front change rates. (c) Use a 30-day median filter to remove outliers from the calving front change time series (blue line). (d) apply a low-pass filter (purple line) to detrend the filtered time series (grey line) in (c). The trend will be used in long-term retreat analysis (gery panel in Figure S1). (e) the detrended calving front change results (black line) from (d), here we apply the LOESS regression to allow better visualization of the seasonal cycles (red line). (f) The seasonal retreat rates for each glacier are calculated as the mean detrended monthly calving front rates in each month over the period of 2014-2023. The blue error bar denotes the most advanced month and the red error bar denotes the most retreated month.

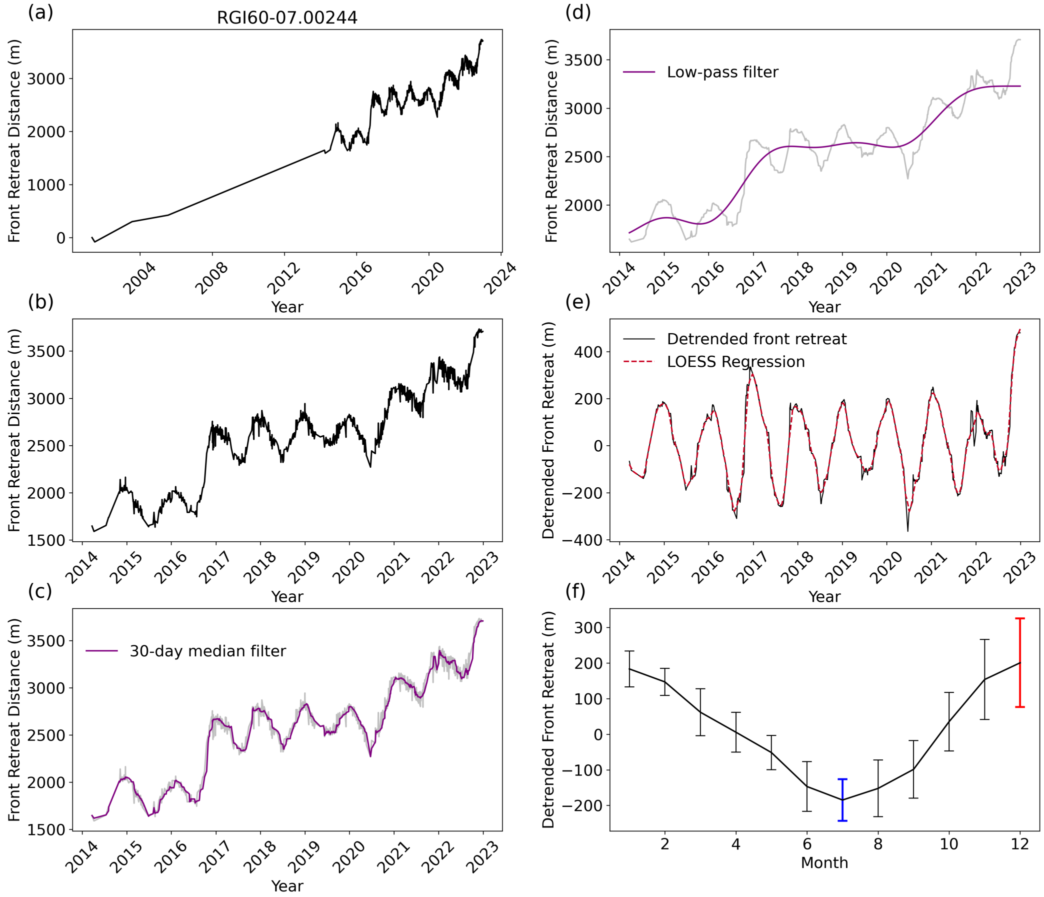

Figure S4: Example of calving front change time series decomposition anlaysis for a retreating glacier Paierlbreen (RGI60-07.00244) following the steps in flowchart (Figure S1). (a) the calving front change time series in 1985-2023. (b) zoomed-in time series in 2014-2023 that is used in calculating the seasonal calving front change rates. (c) Use a 30-day median filter to remove outliers from the calving front change time series (blue line). (d) apply a low-pass filter (purple line) to detrend the filtered time series (grey line) in (c). The trend will be used in long-term retreat analysis (gery panel in Figure S1). (e) the detrended calving front change results (black line) from (d), here we apply the LOESS regression to allow better visualization of the seasonal cycles (red line). (f) The seasonal retreat rates for each glacier are calculated as the mean detrended monthly calving front rates in each month over the period of 2014-2023. The blue error bar denotes the most advanced month and the red error bar denotes the most retreated month.

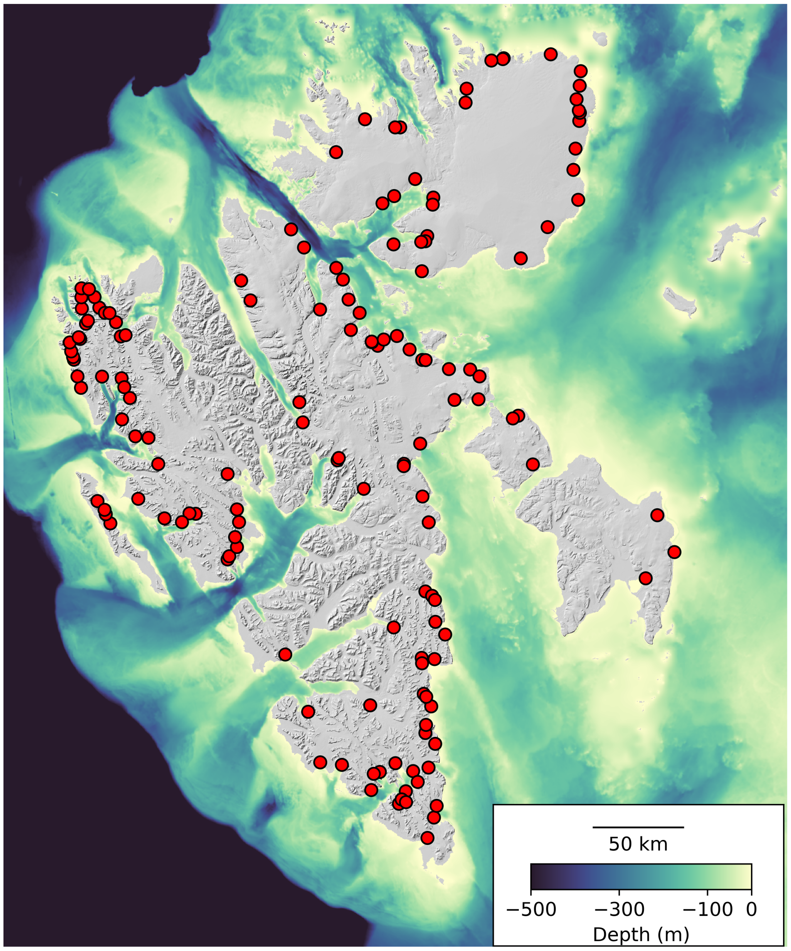

Figure S5: The spatial distribution of glaciers points (red dots) used in deriving surface runoff and 3-m air temperature from the Regional Climate Model Modèle Atmosphérique Régional (MAR) data [2] for all marine-terminating glaciers analyzed in this study. The backgroud ocean bathymetry map is from IBCAO Version 4.2 data [3]. The background hillshade map is generated from the 50 m resolution Svalbard digital elevation model (DEM) (https://data.npolar.no/dataset/dce53a47-c726-4845-85c3-a65b46fe2fea, last access: 18 April 2023).

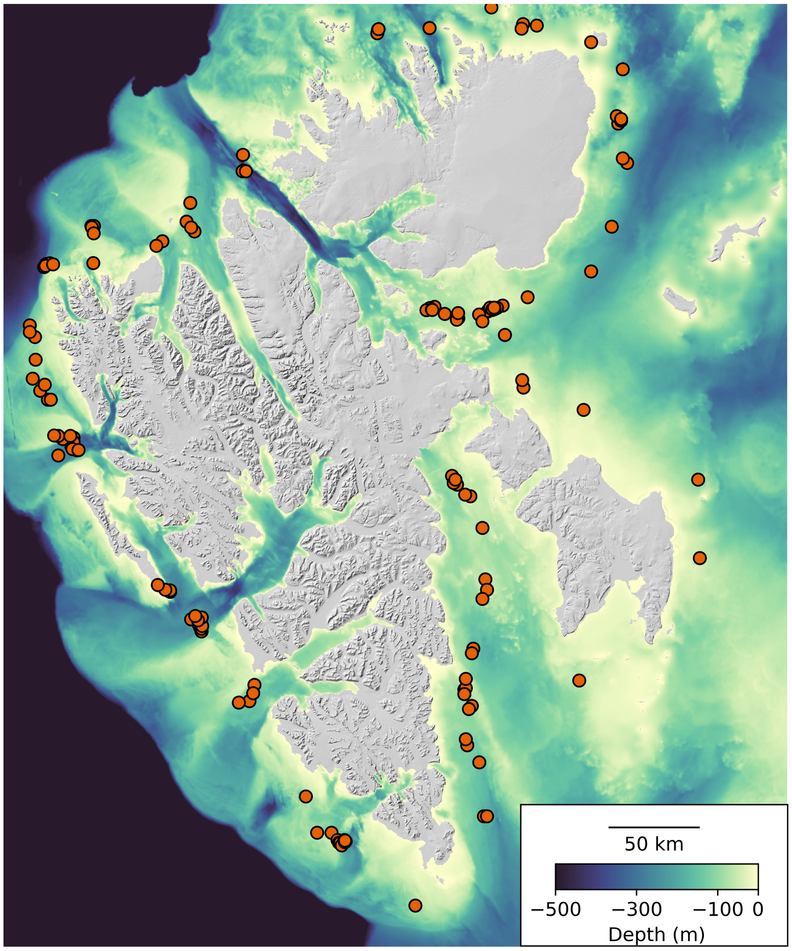

Figure S6: The spatial distribution of fjord points (orange dots) used in deriving sea ice concentrations, 20-100 m depth-averaged subsurface ocean temperature from ECMWF ORAS5 global ocean reanalysis data [4] for all marine-terminating glaciers in this study. The backgroud ocean bathymetry map is from IBCAO Version 4.2 data [3]. The background hillshade map is generated from the 50 m resolution Svalbard digital elevation model (DEM) (https://data.npolar.no/dataset/dce53a47-c726-4845-85c3-a65b46fe2fea, last access: 18 April 2023).

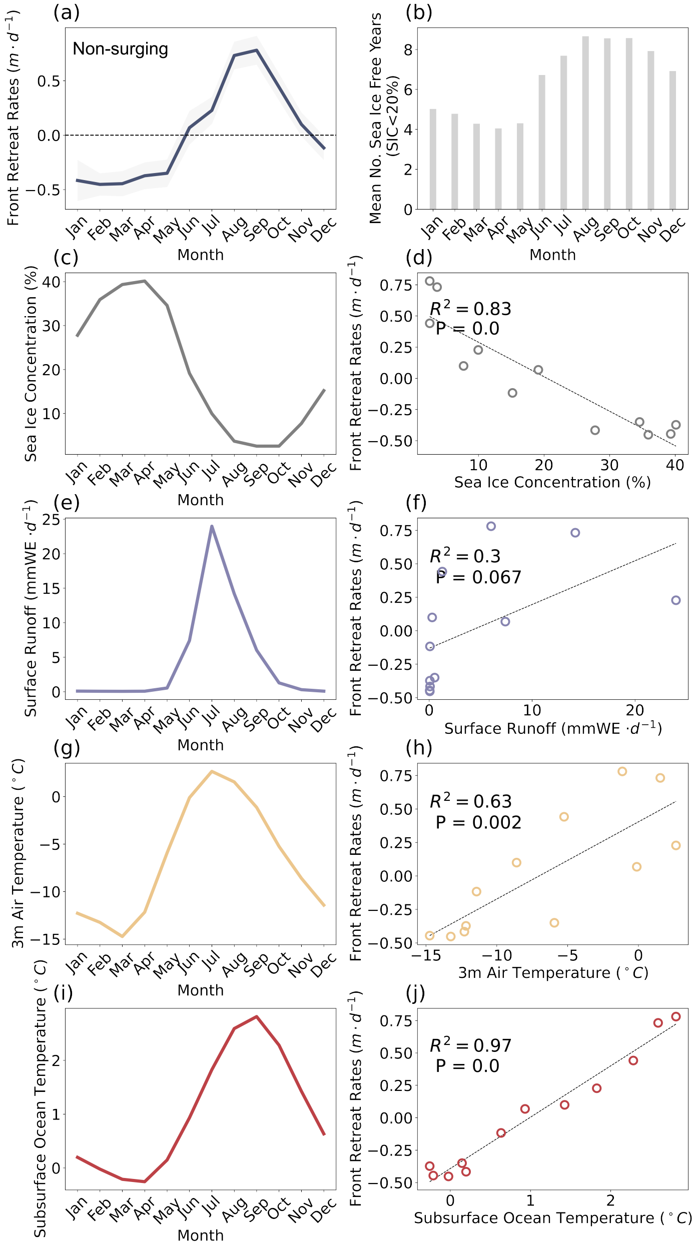

Figure S7: Comparison between mean monthly detrended calving front retreat rates for all 86 non-surging marine-terminating glaciers with seasonal cycles (autocorrelation $>$ 0.08) and different time-evolving environmental variables over the period of 2014-2023. (a) Monthly mean front retreat rates. (b) The number of sea ice free years (when sea ice concentration is less than 20%) in each month. (c) Monthly mean sea ice concentration. (d) Linear regression between monthly mean sea ice concentration and monthly mean retreat rates. (e) Monthly mean surface runoff, and its linear correlation with monthly mean retreat rates (f). (g) Monthly mean 3-m air temperature, and its linear correlation with monthly mean retreat rates (h). (i) Monthly mean subsurface ocean temperature, and its linear correlation with monthly mean retreat rates (j).

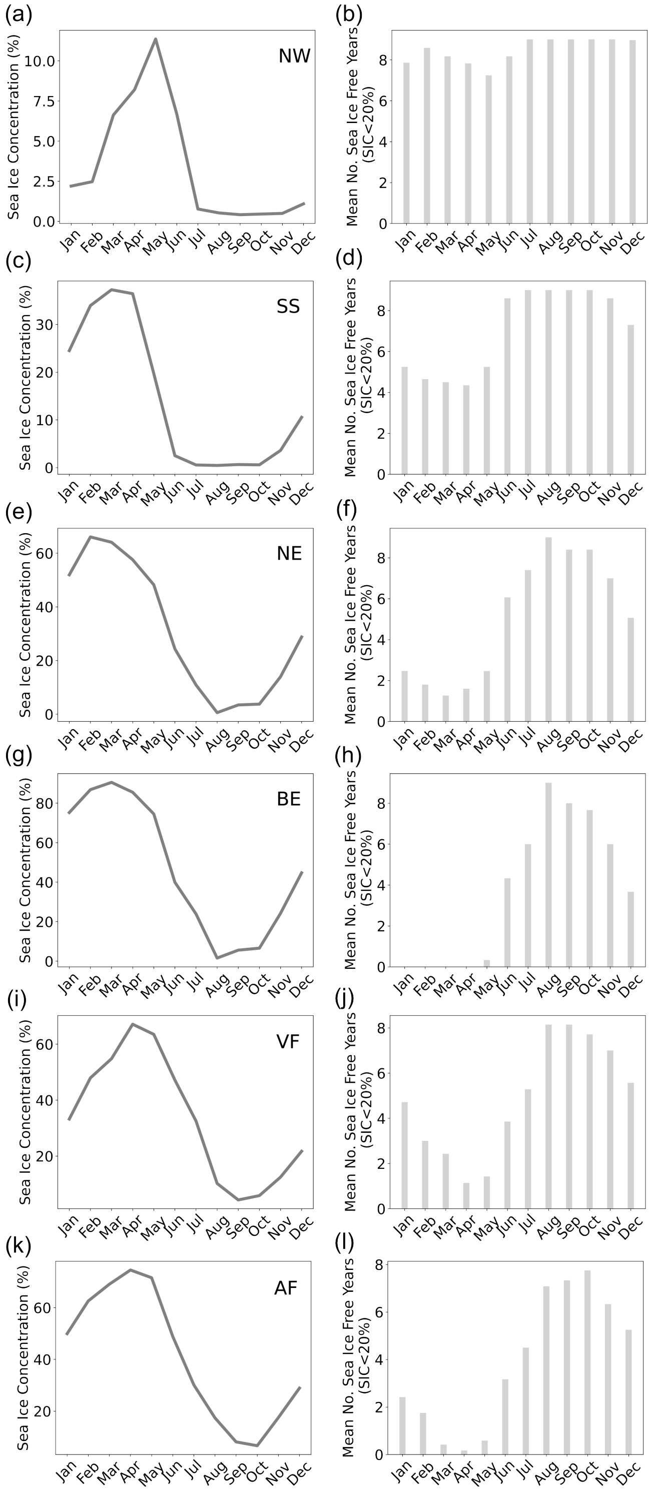

Figure S8: The monthly mean sea ice concentration and the number of sea ice free years (when sea ice concentration is less than 20%) in each month for six different sectors over the period of 2014-2023: Northwest Spitsbergen (a,b), South Spitsbergen (c,d), Northeast Spitsbergen (e,f), Barentsøya and Edgeøya (g,h), Vestfonna (i,j), and Austfonna (k,l).

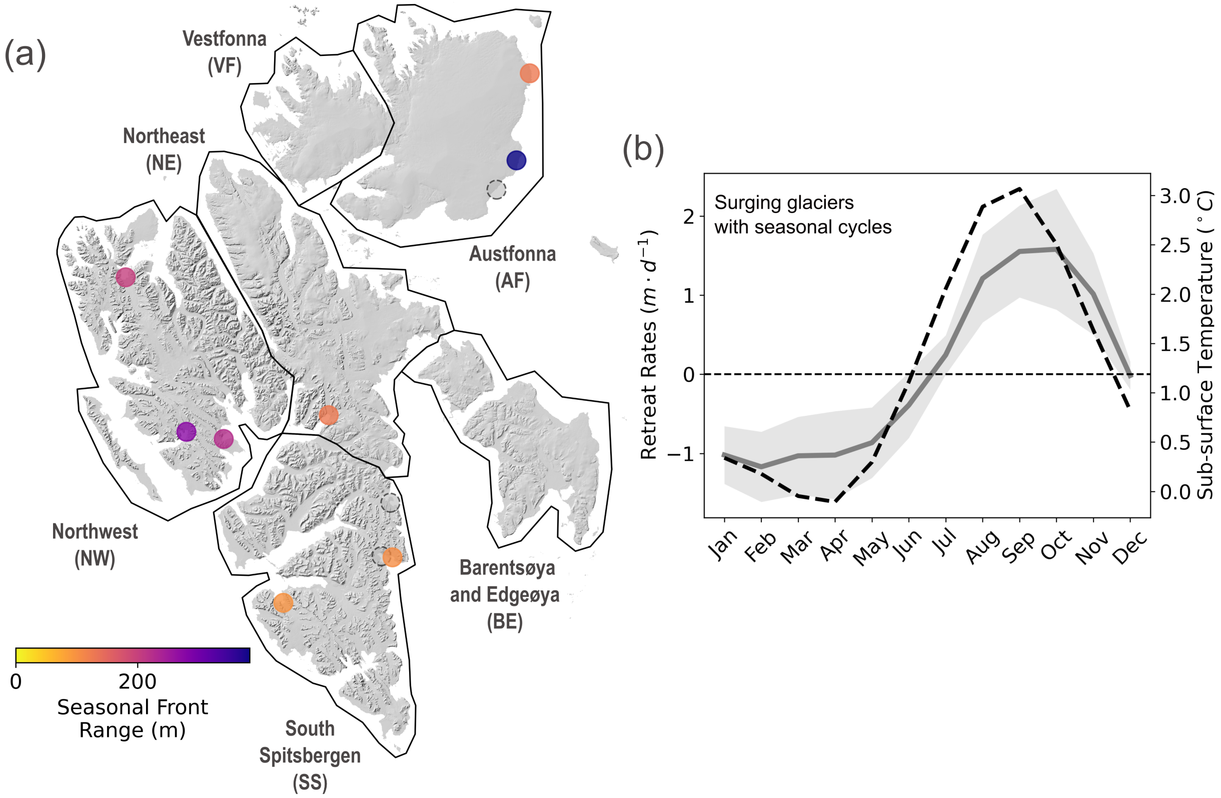

Figure S9: (a) The seasonal calving front range for eight surging glaciers with seasonal cycles (autocorrelation ≥ 0.08). Dashed circles denote the surging glacier with weak or no seasonal cycles. The background hillshade map is generated from the 50 m resolution Svalbard digital elevation model (DEM) (https://data.npolar.no/dataset/dce53a47-c726-4845-85c3-a65b46fe2fea, last access: 18 April 2023). (b) The monthly mean detrended glacier retreat rates for surging glaciers with seasonal cycles across Svalbard (solid grey line) overlaid with monthly mean subsurface ocean temperature (black dashed line).

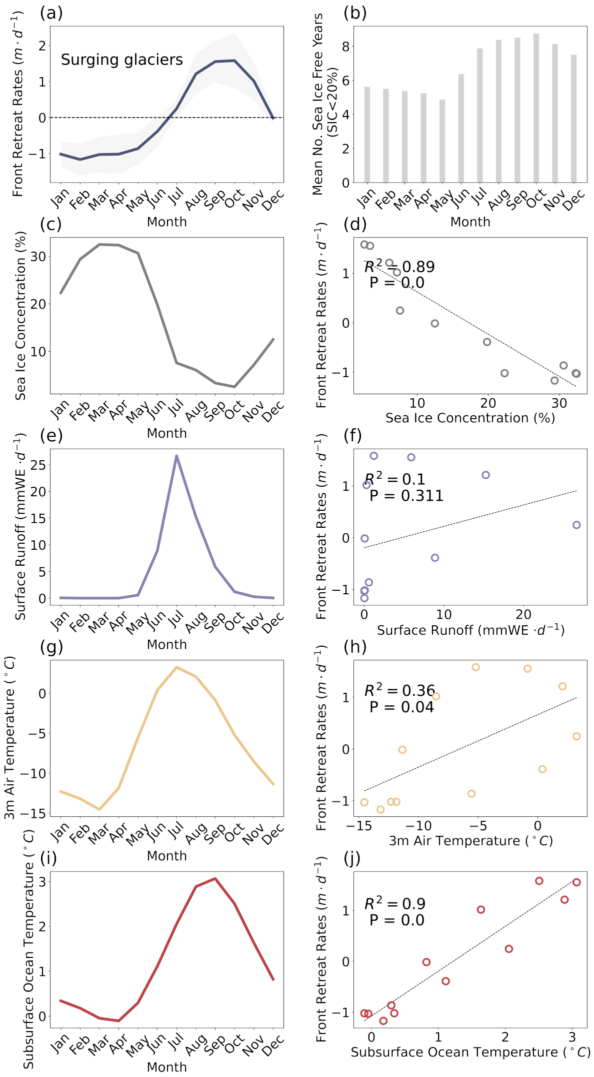

Figure S10: Comparison between mean monthly detrended calving front retreat rates for surging marine-terminating glaciers with seasonal cycles shown in Figure S9a and different time-evolving environmental variables over the period of 2014-2023. (a) Monthly mean front retreat rates. (b) The number of sea ice free years (when sea ice concentration is less than 20%) in each month for 2014-2023. (c) Monthly mean sea ice concentration. (d) Linear regression between monthly mean sea ice concentration and monthly mean retreat rates. (e) Monthly mean surface runoff, and its linear correlation with monthly mean retreat rates (f). (g) Monthly mean 3-m air temperature, and its linear correlation with monthly mean retreat rates (h). (i) Monthly mean subsurface ocean temperature, and its linear correlation with monthly mean retreat rates (j).

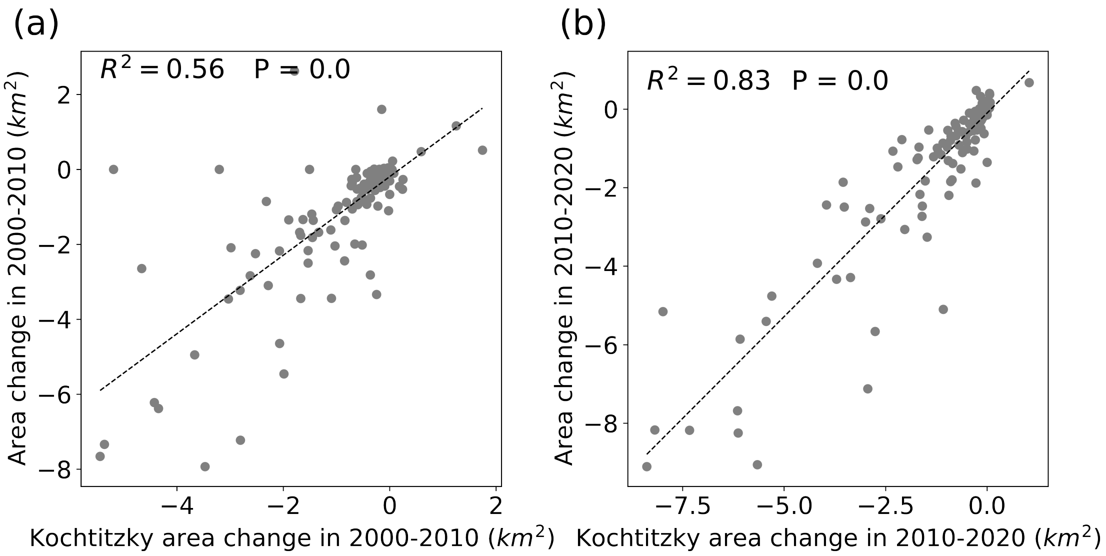

Figure S11: Comparison in areal changes of non-surging marine-terminating glaciers between this study and [5] during two different time periods: (a) 2000-2010; and (b) 2010-2020.

Table S2: The surging glaciers identified in this study (Figure S9) and the period of surging events. The annual periodicity shows the autocorrelation value of along-centerline detrended glacier calving front changes in 2014-2023, strong seasonal change is defined as autocorrelation ≥ 0.08. Sector denotes which region the glacier is located in, including Northeast Spitsbergen (NE), Northwest Spitsbergen (NW), South Spitsbergen (SS) and Austfonna (AF).

Glacier RGI ID	Glacier Name	Surging Period	Annual Periodicity	Sector
RGI60-07.00026	Klerckbukta	2014-2018	0.02	AF
RGI60-07.00027	Storisstraumen	2012-2022	0.08	AF
RGI60-07.00031	Austfonna basin-7	2016-2020	0.16	AF
RGI60-07.00228	Recherchebreen	2018-2020	0.08	SS
RGI60-07.00276	Arnesenbreen	2016-2021	0.02	SS
RGI60-07.00294	Kvalbreen	2020-2022	0.17	SS
RGI60-07.00296	Morsnevbreen	2016-2018	0.04	SS
RGI60-07.00465	Wahlenbergbreen	2015-2018	0.17	NW
RGI60-07.00468	Vestre Osbornebreen	2017-2021	0.3	NW
RGI60-07.01458	Tunabreen	2002-2004 and 2016-2018	0.12	NE
RGI60-07.01494	Monacobreen	2017-2020	0.19	NW

Table S3: Comparison of total area loss in 2000-2020 between this study and [5].

Total Area Change (km^2)	2000-2010	2010-2020	2000-2020
Kochtitzky	180	276	456
This study	262	372	634
Kochtitzky (Remove 5 glaciers in Figure S12)	112	156	268
This study (Remove 5 glaciers in Figure S12)	142	175	317

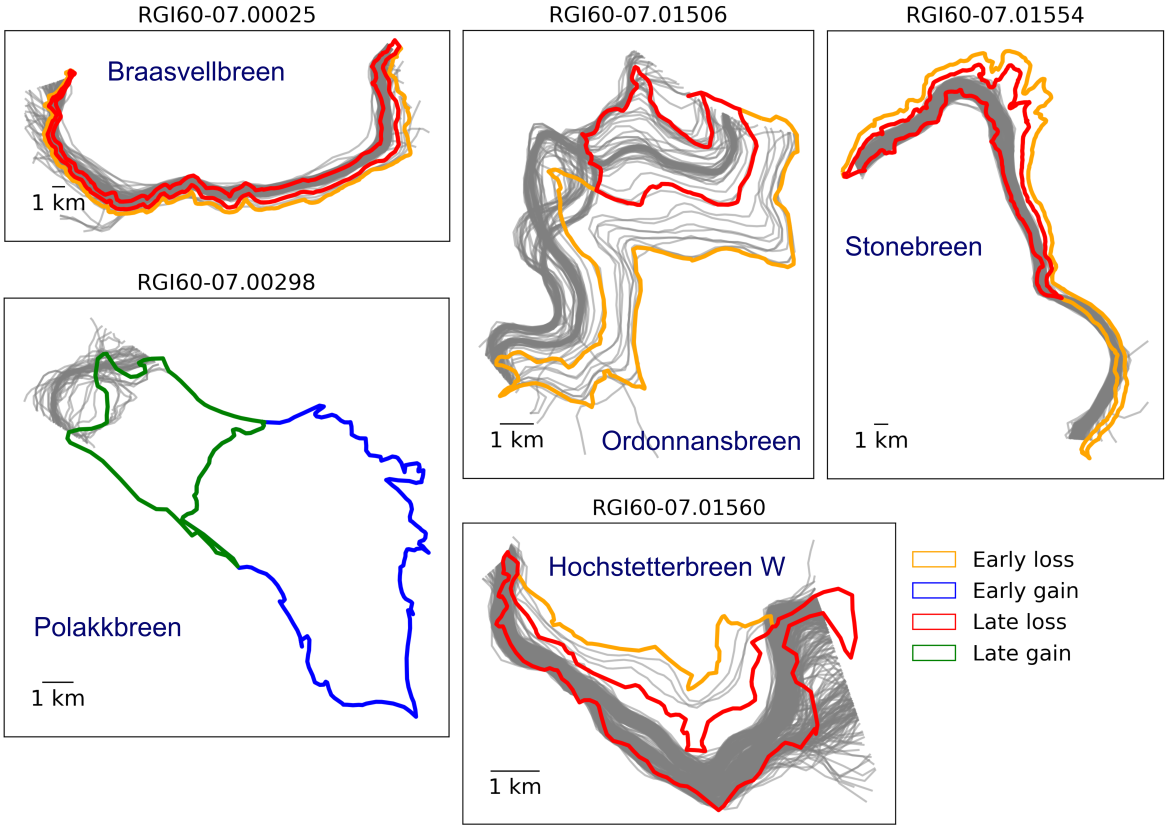

Figure S12: Examples of the five glaciers that have the largest area change differences in 2000-2010 between this study and [5]. The grey lines are the glacier terminus traces generated in [6] over period of 2000-2020, the colored polygons are the areal changes from [5] in different time periods, the 'early loss' means area loss in 2000-2010, the 'early gain' means area increase in 2000-2010, the 'late loss' means area loss in 2010-2020, the 'late gain' means area increase in 2010-2020.

Table S4: Linear correlation coefficients between annual calving front area loss rates and different time-evolving environmental variables in different sectors for non-surging glaciers over period of 1985-2023. Environmental variables include sea ice concentration (SIC), cumulative surface runoff (RU), 3 m air temperature (AT), 20-100 m depth-averaged subsurface ocean temperature (PT). Sectors include including Northeast Spitsbergen (NE), Northwest Spitsbergen (NW), South Spitsbergen (SS), Vestfonna (VF), Austfonna (AF), and Barentsøya and Edgeøya (BE). 'All' means entire Svalbard.

Sector	SIC R^2	SIC P-value	RU R^2	RU P-value	AT R^2	AT P-value	PT R^2	PT P-value
NE	0.25	0.001	0.01	0.606	0.25	0.001	0.42	0
AF	0.2	0.005	0	0.896	0.23	0.002	0.17	0.01
BE	0.12	0.079	0.19	0.022	0.18	0.026	0.14	0.054
NW	0.11	0.042	0.09	0.065	0.17	0.01	0.19	0.06
SS	0.22	0.004	0.3	0.001	0.25	0.002	0.25	0.002
VF	0.05	0.163	0.01	0.546	0.06	0.134	0.1	0.047
All	0.34	0	0.08	0.078	0.39	0	0.5	0

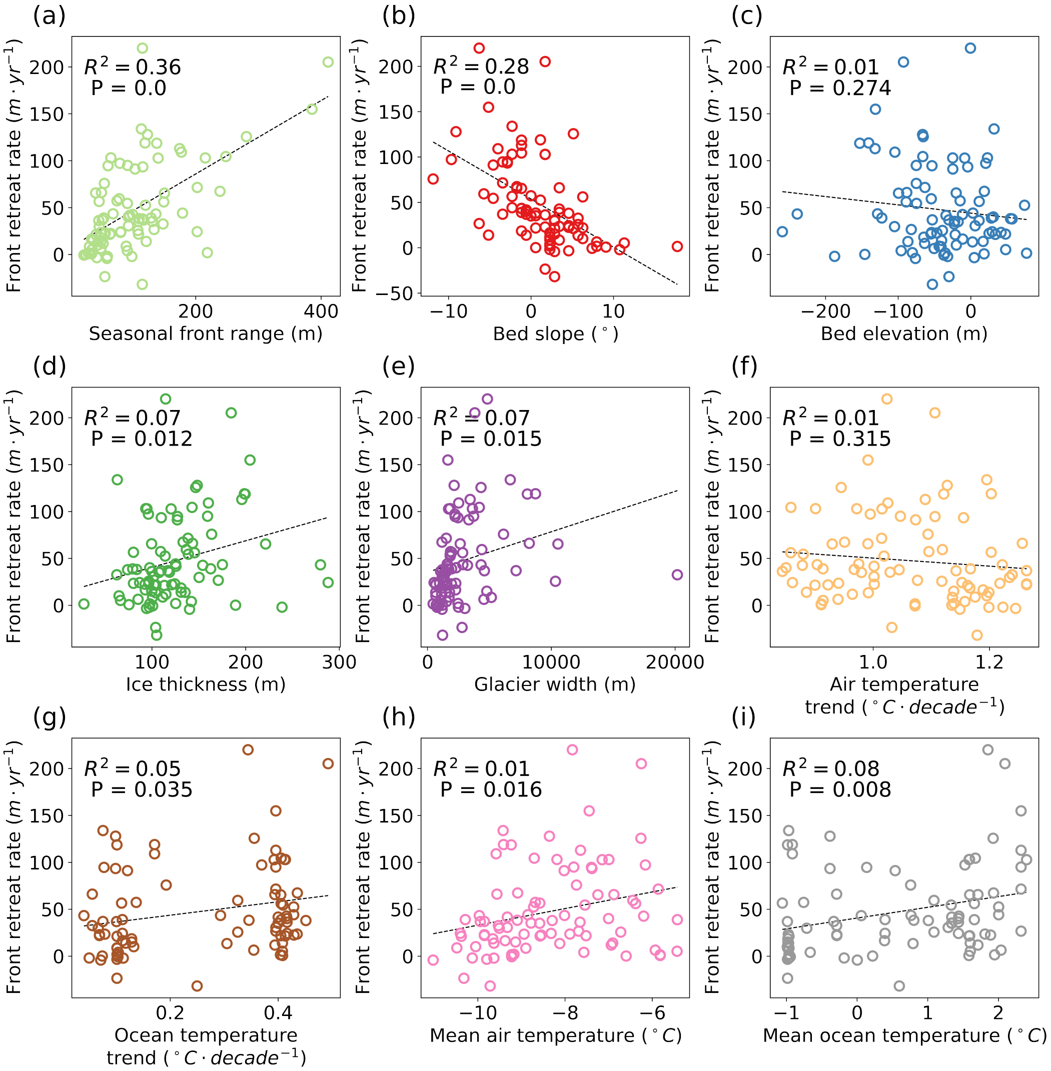

Figure S13: Decadal front retreat rates for 86 non-surging glaciers with seasonal cycles and their linear correlations with 9 predictors of sensitivity to climate change. The strongest correlations are found for seasonal front range (a) and bed slope (b). The bed slope, bed elevation (c), ice thickness (d) are derived from SVIFT bed topography data [7] at calving fronts (see Online Methods). Glacier width (e) is calculated as the mean length of all available terminus traces for each glacier. Decadal trend in air temperature (f) and subsurface ocean temperature (g) are calculated by fitting a trend line from the 1985-2023 temperature time series of each glacier. Mean air temperature (h) and mean subsurface ocean temperature (i) are calculated as the mean temperatures from the 1985-2023 temperature time series of each glacier.

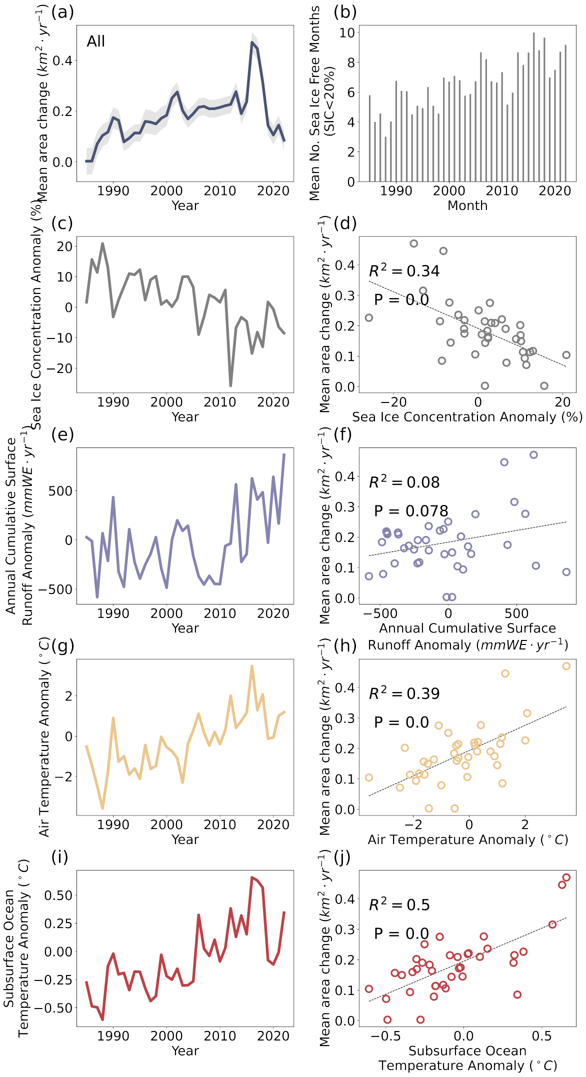

Figure S14: Comparison between annual calving front area loss rates for all non-surging marine-terminating glaciers and different time-evolving environmental variables. (a) Annual mean calving front area loss rates. (b) The number of sea ice free months (when sea ice concentration is less than 20%) in each year. (c) Annual mean sea ice concentration anomaly. (d) Linear regression between annual mean sea ice concentration anomaly and annually mean calving front area loss rates. (e) Annual cumulative surface runoff anomaly, and its linear correlation with calving front area loss rates (f). (g) Annual 3-m air temperature anomaly, and its linear correlation with calving front area loss rates (h). (i) Annual subsurface ocean temperature anomaly, and its linear correlation with calving front area loss rates (j).

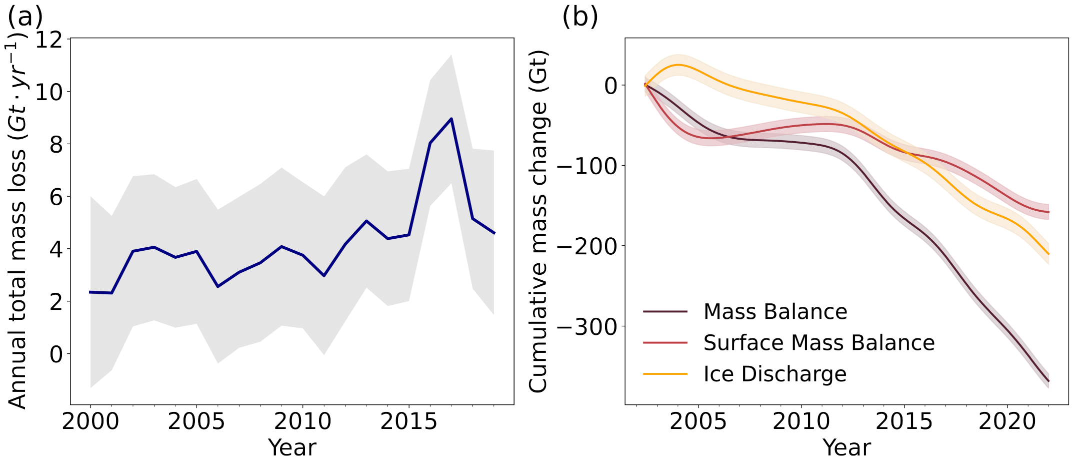

Figure S15: Mass loss in Svalbard over the past two decades. (a) Annual total mass loss rates for non-surging marine-terminating glaciers in Svalbard between 2000 and 2020 [8]. (b) Cumulative mass change of Svalbard glaciers calculated from GRACE JPL Mascons and cumulative surface mass balance calculated from CARRA Copernicus Arctic Regional Reanalysis data [9], as well as cumulative ice discharge estimated from the difference between total mass balance and surface mass balance.

References

[1] T. Li, K. Heidler, L. Mou, Á. Ignéczi, X. X. Zhu, and J. L. Bamber. A high-resolution calving front data product for marine-terminating glaciers in svalbard. *Earth System Science Data*, 16(2):919–939, 2024. doi: 10.5194/essd-16-919-2024.

[2] X. Fettweis, B. Franco, M. Tedesco, J. H. van Angelen, J. T. M. Lenaerts, M. R. van den Broeke, and H. Gallée. Estimating the greenland ice sheet surface mass balance contribution to future sea level rise using the regional atmospheric climate model mar. *The Cryosphere*, 7(2):469–489, 2013. doi: 10.5194/tc-7-469-2013.

[3] M. Jakobsson, L. Mayer, B. Coakley, J. A. Dowdeswell, S. Forbes, B. Fridman, H. Hodnesdal, R. Noormets, R. Pedersen, M. Rebesco, H. W. Schenke, Y. Zarayskaya, D. Accettella, A. Armstrong, R. M. Anderson, P. Bienhoff, A. Camerlenghi, I. Church, M. Edwards, J. V. Gardner, J. K. Hall, B. Hell, O. Hestvik, Y. Kristoffersen, C. Marcussen, R. Mohammad, D. Mosher, S. V. Nghiem, M. T. Pedrosa, P. G. Travaglini, and P. Weatherall. The international bathymetric chart of the arctic ocean (ibcao) version 3.0. *Geophysical Research Letters*, 39(12), 2012. ISSN 1944-8007. doi: 10.1029/2012gl052219.

[4] H. Zuo, M. A. Balmaseda, S. Tietsche, K. Mogensen, and M. Mayer. The ecmwf operational ensemble reanalysis-analysis system for ocean and sea ice: A description of the system and assessment. *Ocean Science*, 15:779–808, 2019.

[5] W. Kochtitzky and L. Copland. Retreat of northern hemisphere marine-terminating glaciers, 2000–2020. *Geophysical Research Letters*, 49:e2021GL096501, 2022.

[6] T. Li, K. Heidler, L. Mou, Á. Ignéczi, X. X. Zhu, and J. Bamber. Calving front dataset for marine-terminating glaciers in svalbard 1985-2023 [data set]. *Zenodo*, 2023. doi: https://doi.org/10.5281/zenodo.10407266.

[7] J. J. Fürst, F. Navarro, F. Gillet-Chaulet, M. Huss, G. Moholdt, X. Fettweis, C. Lang, T. Seehaus, S. Ai, T. J. Benham, D. I. Benn, H. Björnsson, J. A. Dowdeswell, M. Grabiec, J. Kohler, I. Lavrentiev, K. Lindbäck, K. Melvold, R. Pettersson, D. Rippin, A. Saintenoy, P. Sánchez-Gámez, T. V. Schuler, H. Sevestre, E. Vasilenko, and M. H. Braun. The ice-free topography of svalbard. *Geophysical Research Letters*, 45(21):11,760–11,769, 2018. doi: https://doi.org/10.1029/2018GL079734.

[8] R. Hugonnet, R. McNabb, E. Berthier, B. Menounos, C. Nuth, L. Girod, D. Farinotti, M. Huss, I. Dussaillant, F. Brun, and A. Kääb. Accelerated global glacier mass loss in the early twenty-first century. *Nature*, 592(7856):726–731, 2021. doi: 10.1038/s41586-021-03436-z.

[9] L. S. Schmidt, T. V. Schuler, E. E. Thomas, and S. Westermann. Meltwater runoff and glacier mass balance in the high arctic: 1991-2022 simulations for svalbard. *Cryosphere*, 17:2941–2963, 2023.
